# Genetic Diversity and Connectivity in *Maurolicus muelleri* in the Bay of Biscay Inferred from Thousands of SNP Markers

**DOI:** 10.3389/fgene.2017.00195

**Published:** 2017-11-28

**Authors:** Naiara Rodriguez-Ezpeleta, Paula Álvarez, Xabier Irigoien

**Affiliations:** ^1^Marine Research Division, AZTI, Sukarrieta, Spain; ^2^Marine Research Division, AZTI, Pasaia, Spain

**Keywords:** mesopelagic fish, high-throughput sequencing, RAD-seq, population connectivity, genetic diversity, *Maurolicus muelleri*

## Abstract

Mesopelagic fish are largely abundant poorly studied fish that are still intact, but which, due to their potentially great added value, will be imminently exploited by humans. Therefore, studies that provide information to anticipate the anthropogenic impact on this important resource are urgently needed. In particular, knowledge about their connectivity, potential adaptation and resilience are needed. This information can be obtained through the analysis of genome-wide markers which are now relatively easily and cost-efficiently discovered thanks to high-throughput sequencing technologies. Here, we have generated thousands of SNP markers in *Maurolicus muelleri*, based on the restriction-site associated DNA sequencing method, and preformed population connectivity and genetic diversity analyses in a subset of samples collected from the Bay of Biscay. Our study proves the method valid for obtaining genome-wide markers in this species and provides the first insights into the population genomics of *M. muelleri*. Importantly, the genomic resources developed here are made available for future studies and set the basics for additional endeavors on this issue.

## Introduction

The lack of physical barriers in the ocean and large populations sizes generally results in low genetic differentiation in marine fishes (Ward et al., 1994; Bradbury et al., 2008), which renders the task of inferring demographic patterns in this environment particularly difficult. Yet, over the last few years, an increasing number of studies based on high-throughput genetic data have provided evidences of fine scale population differentiation (e.g., Hess et al., 2013; Benestan et al., 2015; Rodríguez-Ezpeleta et al., 2016), challenging previous assumptions based on traditional methods (Hauser and Carvalho, 2008). In particular, the advent of the restriction site associated DNA sequencing (RAD-seq) (Baird et al., 2008) and related methods (Davey et al., 2011) has revolutionized the field of marine conservation genomics (Narum et al., 2013). This approach consists on subsampling putative homologous regions from the genome in several individuals with the aim of identifying and genotyping single nucleotide polymorphisms (SNPs). Interestingly, the method can be applied to organisms for which no prior genomic resources are available, and it is suitable to study both, neutral population structure and local adaptation (Allendorf et al., 2010). In the context of marine management, RAD-seq is particularly relevant for poorly studied widely distributed species, as it can provide quick estimates of genetic diversity, population connectivity and adaptation more cost-effectively than relying on genome or transcriptome sequencing, or on non-sequencing based microsatellite or SNP typing.

Among the poorly studied marine organisms are mesopelagic fish, i.e., those inhabiting the deep scattering layer, that represent the largest fish biomass in the ocean (Kaartvedt et al., 2012; Irigoien et al., 2014) and play important roles in the marine ecosystem and global biogeochemical cycles (Klevjer et al., 2016). The large abundance of these fish make them particularly attractive for exploitation (St. John et al., 2016). Yet, the mesopelagic ecosystem is still largely unknown (Sutton, 2013), and the potential effects of newly introduced anthropogenic pressures in this realm should be anticipated so that sustainable management strategies for this valuable resource can be developed (St. John et al., 2016). Thus, there is an urgent need for establishing focused scientific surveys, developing appropriate sampling gear and generating additional biological data to booster knowledge in these species. In particular, developing genomic tools is foremost, as they can provide insights to understand population persistence, productivity and response to exploitation (Palumbi, 2003; Cowen et al., 2006).

Several studies have focused on deciphering species boundaries and population connectivity within mesopelagic fishes using genetic variants, but they are all based on allozymes (Gunawickrama and Naevdal, 2001; Kristoffersen and Salvanes, 2009), single mitochondrial markers (Kojima et al., 2009; Habib et al., 2012; Gordeeva, 2014) or a few microsatellites (Gordeeva, 2011; Van de Putte et al., 2012). To our knowledge, no studies based on genomic-wide SNP data have been published. Yet, simulated and empirical data based evidences support that high-throughput SNP data analyses provide more accurate population structure inferences than single or a few polymorphic marker based analyses (Haasl and Payseur, 2011; Rašić et al., 2014; Benestan et al., 2015; Szulkin et al., 2016). Here, in a first attempt to introduce genome-wide SNP discovery and genotyping to study mesopelagic fishes, we have focused on the Mueller’s pearlside, *Maurolicus muelleri.*

Although initially thought to be distributed worldwide, previous reports of *M. muelleri* in the Northeast Atlantic (Gjøsætr and Kawaguchi, 1980), the South Atlantic (Hulley and Prosch, 1987), the Southeast Pacific (Robertson, 1976), and the coasts of Japan (Okiyama, 1971) are now believed to belong to different species of the genus *Maurolicus*. The genus was split into fifteen species based on morphological characters and geographic distribution (Parin and Kobyliansky, 1993), but recent studies have suggested an overestimate of species diversity in *Maurolicus* (Kim et al., 2008; Rees et al., 2017), illustrating a need for more in-depth phylogeographical analyses. In response to these needs, we have discovered and genotyped thousands of SNP markers in *M. muelleri*, which constitute the first genomic resource of a mesopelagic fish.

## Materials and Methods

### Tissue Sampling and DNA Extraction

**FIGURE 1:**
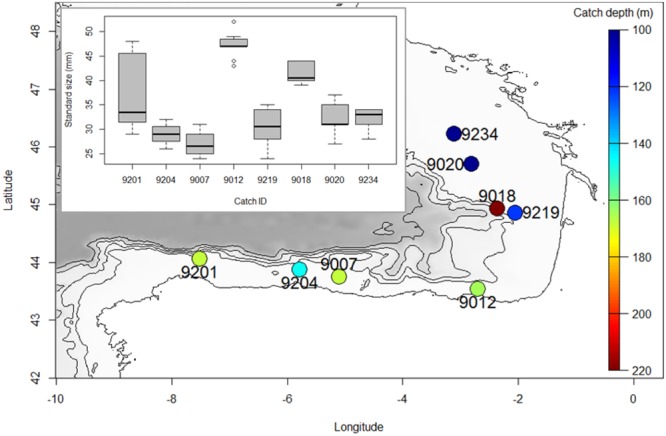
Map depicting the location where samples used for this study were obtained. Color indicates depth at which samples were caught, and boxplots depict size distribution of each catch.

Specimens of *M. muelleri* were collected at several coastal locations of southern Bay of Biscay (**Figure 1**) through pelagic trawling on board the R/V Emma Bardán and Ramón Margalef in September 2016. Catches were collected between 106 and 212 m and during daytime. Fish where frozen at -20°C until DNA extraction. Genomic DNA was extracted from about 20 mg of tissue using the Wizard^®^ Genomic DNA Purification kit (Promega, Fitchburg, WI, United States) following manufacturer’s instructions for “Isolating Genomic DNA from Tissue Culture Cells and Animal Tissue.” Extracted DNA was suspended in Milli-Q water and concentration was determined with the Quant-iT dsDNA HS assay kit using a Qubit^®^ 2.0 Fluorometer (Life Technologies). DNA integrity was assessed by electrophoresis, migrating about 100 ng of GelRed^TM^-stained DNA on an agarose 1.0% gel.

### RAD-Seq Library Preparation and Data Analysis

Restriction-site-associated DNA libraries of 94 individuals (10 to 20 per catch) were prepared following the methods of Etter et al. (2011). Briefly, starting DNA (ranging from 300 to 500 ng) was digested with the *SbfI* restriction enzyme and ligated to modified Illumina P1 adapters containing 5 bp unique barcodes. Pools of 30 or 32 individuals were sheared using the Covaris^®^ M220 Focused-ultrasonicator^TM^ Instrument (Life Technologies) and size selected to 300–500 pb by cutting agarose migrated DNA. After Illumina P2 adaptor (including 5 pb index) ligation, each library was amplified using 14 PCR cycles. The three pools, each with one unique Illumina index, were combined and paired-end sequenced (100 pb) on an Illumina HiSeq2000. Generated RAD-tags were analyzed using *Stacks* version 1.44 (Catchen et al., 2013b). Quality filtering and demultiplexing was performed with *process_radtags* with default parameters and removing the last 10 bases of the end of the reads. Putative orthologous tags (stacks) per individual were assembled using *ustacks* with a minimum depth of coverage required to create a stack (m) of 3 and a maximum nucleotide mismatches (M) allowed between stacks of 2, 4, or 6. Catalogs of RAD loci were assembled using *cstacks* with number of mismatches allowed between sample tags when generating the catalog (n) of 3, 6, and 9 for *M*-values of 2, 4, and 6, respectively. Matches of individual RAD loci to the catalog were searched using *sstacks*. RAD loci found in at least 75% of the individuals were selected using *populations* and used to calculate (nucleotide diversity – π, minor allele frequency – MAF, expected heterozygosity – *H*_e_, expected homozygosity – *H*_o_, and inbreeding coefficient – *F*_IS_). Using *PLINK* version 1.07 (Purcell et al., 2007), SNPs with MAF larger than 0.05 and a genotyping rate larger than 0.9 were selected for population structure analyses. The obtained genotype dataset was exported to *Structure* and *Genepop* formats using *PGDSpider* version 2.0.8.3 (Lischer and Excoffier, 2012).

### Genetic Diversity and Population Structure

*F*_ST_ values per group pairs were calculated following the Weir and Cockerham (1984) formulation as implemented in *Genepop 4.3* (Rousset, 2008). Principal component analyses (PCA) were performed with the R package *adegenet* (Jombart and Ahmed, 2011) without any *a priori* grouping assumption. The percentage of appurtenance of each individual to each of the *K* (ranging from 1 to 4) hypothetical ancestral was calculated with the Bayesian clustering approach implemented in *STRUCTURE* (Pritchard et al., 2000) without any prior population assignment, based on the admixture model and a burn-in period of 100,000 iterations followed by 300,000 iterations from which estimates were obtained. Ten replicates for each *K*-value were performed and analyzed with *CLUMPP* (Jakobsson and Rosenberg, 2007) to identify common modes. Results were plotted using *DISTRUCT* (Rosenberg, 2004), and best *K* was identified according to the Evanno method (Evanno et al., 2005) as implemented in *StructureHarvester* (Earl and vonHoldt, 2012).

## Results

### RAD-Seq Genotyping

**FIGURE 2:**
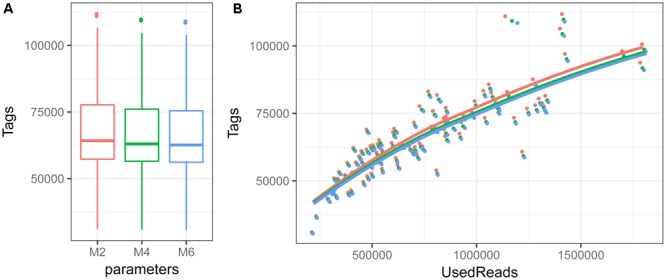
Boxplot depicting first and third quartile, median and standard deviation of number of RAD-tags per individual **(A)** and relationship among number of RAD-tags and used reads **(B)** when the *M* parameter is set to 2 (red), 4 (green) or 6 (blue).

The number of RAD-seq reads passing quality filters per individual included in the final analyses ranges from 326 824 to 2 116 073 with an average of 974 008. The number of RAD-tags obtained per individual ranges from about 30K to about 110K, with an average of 67 592, 66 359, and 65 852 for *M*-values of 2, 4, and 6, respectively (**Figure 2A**). Average coverage per individual ranges from 6 to 20× (with an average of 11×). Number of RAD-tags per individual increases with number of reads used for stack formation and does not reach a maximum value even when more than 100K reads are used (**Figure 2B**). This places the number of *SbfI* cut sites in *M. muelleri* above 32 000, which is larger than in other teleost species (Catchen et al., 2013a; Diaz-Arce et al., 2016; Rodríguez-Ezpeleta et al., 2016).

Above twenty thousand RAD-tags are present in at least 75% of the individuals, and comprise more than two million sites of which more than a thousand are variable (**Table 1**). The number of tags present in more than 75% of the individuals increases with increasing *M* because more common loci can be found when these are composed by more alleles. Consequently, larger *M*-values results in more alleles and thus more variable positions (Catchen et al., 2013b; Rodríguez-Ezpeleta et al., 2016). Selecting only those positions with MAF larger than 0.05 and applying a stringent filtering of a minimum of 90% of the individuals being genotyped per SNP, results in three genotype datasets of 1 625, 2 350, and 2 409 SNPs for *M*-values 2, 4, and 6, respectively.

**Table 1:** Number of tags present in at least 75% of the individuals, as well as number of positions remaining after each filtering step for each value of *M* used.

	*M* = 2	*M* = 4	*M* = 6
Number of tags present in >75% of individuals	22 624	24 179	24 504
Number of positions	2 031 164	2 166 128	2 190 124
Number of variable positions (SNPs)	132 782	180 398	207 080
Number of SNPs with MAF >0.05	13 852	19 925	22 331
Number of SNPs genotyped in >90% of individuals	1 625	2 350	2 409

### Genetic Diversity

**FIGURE 3:**
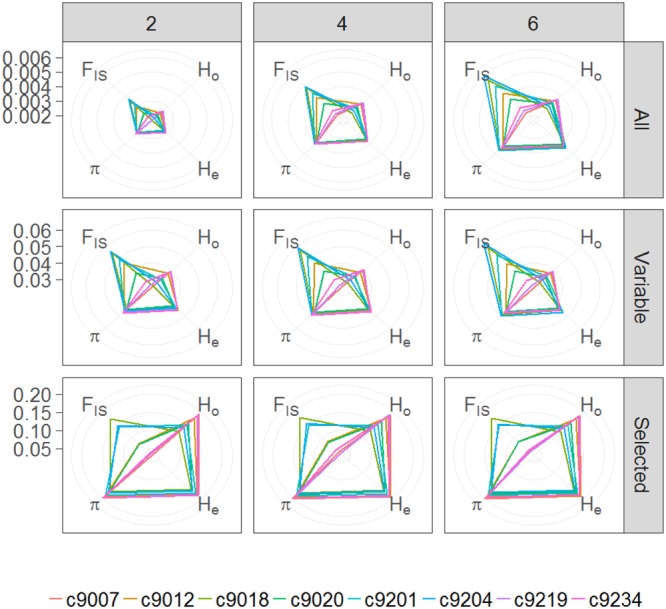
Radar plots showing average nucleotide diversity (π), inbreeding coefficient (*F*_IS_), and expected (*H*_e_) and observed (*H*_o_) heterozygosity for each catch (colored lines) considering each value of *M* (2, 4, or 6) and for either, all positions included in tags present in at least 75% of the individuals (All), only variable positions within these tags (Variable) or only selected SNPs (Selected).

The number of polymorphic sites contained in the tags present in at least 75% off the individuals is 6.5, 8.3, and 9.4% for *M*-values of 2, 4, and 6, respectively. When calculated considering all individuals a single group, overall nucleotide diversity (π), inbreeding coefficient (*F*_IS_), and expected (*H*_e_) and observed heterozygosity (*H*_o_) values are congruent across the three parameter sets used. Expectedly, using only variable sites or selecting those with at least 0.05 MAF increases nucleotide diversity and both, expected and observed heterozygosity (**Table 2**). For all set of parameters and position subsets used, observed heterozygosity is lower than expected both overall and when calculated per catch. Per catch, values of π and *H*_e_ are similar, whereas values of *H*_o_ and *F*_IS_ differ (**Figure 3**). In general, catches with low observed heterozygosity are also those with highest inbreeding coefficient (9018, 9204, 9201) and vice versa (hauls 9007, 9219, 9234), although this does not hold for catches 9012 and 9029 with present large *H*_o,_ but medium *F*_Is_ values with respect to the others. Similar to other studies (Rodríguez-Ezpeleta et al., 2016), we find that, for the four variables, absolute values depend on the set of parameters used for SNP discovery, but that relative values among groups are maintained.

**Table 2:** Average nucleotide diversity (π), inbreeding coefficient (*F*_IS_), and observed (*H*_o_) and expected (*H*_e_) heterozygosity for each value of *M* when considering all positions included in tags present in at least 75% of the individuals (All), only variable positions within these tags (Variable) or only selected SNPs (Selected).

*M*		π	*F*_IS_	*H*_o_	*H*_e_
2	All	0.0030	0.0105	0.0023	0.0030
	Variable	0.0465	0.1613	0.0350	0.0462
	Selected	0.1981	0.1458	0.1652	0.1880
4	All	0.0040	0.0135	0.0030	0.0040
	Variable	0.0487	0.1632	0.0364	0.0484
	Selected	0.1936	0.1537	0.1651	0.1925
6	All	0.0045	0.0163	0.0034	0.0045
	Variable	0.0482	0.1741	0.0359	0.0479
	Selected	0.1911	0.1543	0.1616	0.1900

### Population Structure

**FIGURE 4:**
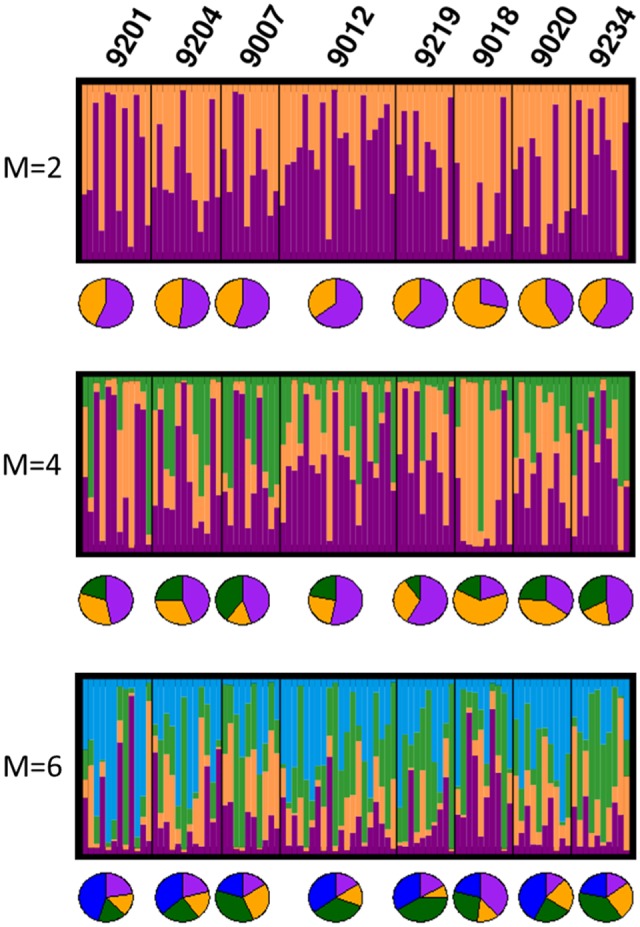
Graphical representation of the Bayesian clustering approach for the best *K*-value obtained for *M*-values 2, 4, or 6; each bar represents an individual and each color, its inferred membership to each *K* potential ancestral populations. Pie charts represent per catch averaged proportion of assignment to each potential ancestral population. For each *M*-value, only the best value of *K* is shown.

**FIGURE 5:**
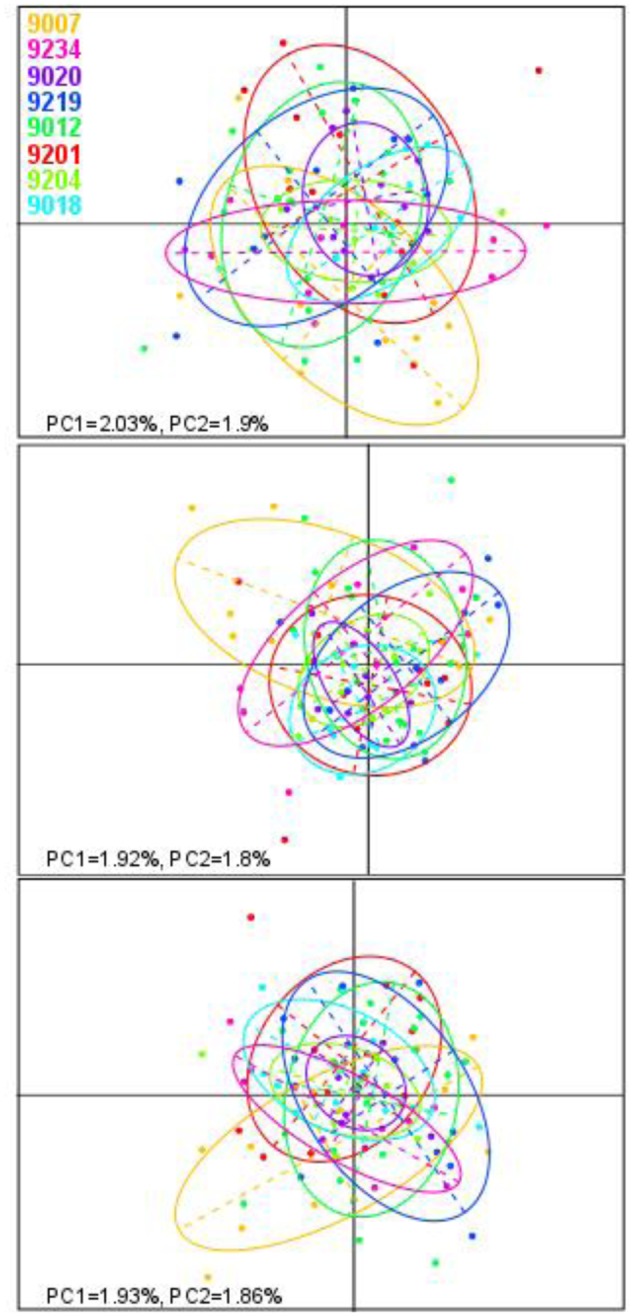
Principal component analysis (PCA) of allele frequencies. Each plot shows the first two principal components of the PCA obtained from datasets built using *M*-values of 2, 4, or 6 (top to bottom). Each dot represents one sample and is colored according to the area of origin. Ovals represent 95% inertia ellipses.

In the Bayesian population structure analyses (**Figure 4**), all individuals display admixed representation of each of the hypothetical ancestral population, suggesting genetic connectivity within the area of study. Interestingly, catch 9018 shows an ancestry pattern that differs from the rest of the catches, yet, this difference is not visible in the PCA plots (**Figure 5**), where no differentiation among groups can be observed. In accordance with the hypothesis of high connectivity within the area of study, *F*_ST_ values between pairs of populations are low (**Table 3**), although, consistent with the pattern observed in the structure analyses, pairs including catch 9018 are those with highest *F*_ST_ values.

**Table 3:** *F*_ST_ values per population pair.

9201	9201	9204	9007	9012	9219	9018	9020
9204	0.000						
9007	0.000	0.003					
9012	0.000	0.005	0.000				
9219	0.004	0.011	0.008	0.003			
9018	0.002	0.008	0.009	0.010	0.005		
9020	0.001	0.005	0.006	0.004	0.007	0.003	
9234	0.002	0.004	0.000	0.001	0.002	0.004	0.003

*The three *M* parameter values tested provided congruent outcomes; here, only those values corresponding to *M* = 2 are shown.*

## Discussion

Restriction-site associated DNA sequencing constitutes an unprecedented opportunity for performing demographic inferences in species for which no prior genetic resources are available (Corander et al., 2013; Hess et al., 2013). Here, we have discovered and genotyped thousands of RAD-seq derived SNP markers in *M. muelleri* in a first attempt to use genome wide data to study diversity and connectivity in this mesopelagic species, which is particularly relevant in view of the imminent exploitation of this till now pristine marine resource.

We have demonstrated that the *Sbf I* restriction enzyme is as a good candidate for RAD-sequencing based SNP discovery in *M. muelleri.* Interestingly, we found that, although the average number of RAD-tags per individual is similar to that found in other species (Amores et al., 2011; Catchen et al., 2013b; Puebla et al., 2014; Diaz-Arce et al., 2016; Rodríguez-Ezpeleta et al., 2016), the number of RAD-tags obtained per individual increases with the number of sequencing reads produced; this suggests that most likely the genome of *M. muelleri* could have as many as 50 000 *SbfI* cut sites, which is higher than most fish species studied so far. The presence of a large number of cut sites, and therefore of loci, could suggest lower coverage per locus. Yet, although lower coverage than in other species is obtained, all individuals had more than 6× coverage and average was of 11×. Multiplexing less individuals per lane or sequencing the same pool in more than one lane would increase coverage accordingly. Yet, the large number of loci shared among 75% of the individuals and the above one thousand SNP markers obtained after filtering suggest that this increased sequencing would not be necessary.

In our analyses, observed heterozygosity is lower than expected. This could be indicator of (i) population differentiation within the area of study, referred to as the Wahlund effect (Wahlund, 1928), (ii) preferential mating with close relatives or inbreeding (Wright, 1921) or (iii) non-random sampling of members from a limited number of families (Robertson, 1965). Due to the large biomass of mesopelagic fish (Irigoien et al., 2014), it would seem unlikely that our samples, composed by a few individuals per location, contain relatives. Similarly, preferential mating with close relatives or inbreeding would be difficult also to explain. Thus, the natural explanation for the low observed heterozygosity obtained would be population differentiation within the area of study. Yet, we do not find clear evidences of population stratification except for catch 9018, which appears genetically differentiated in the STRUCTURE plots, but not in the PCA.

The fact that when separating individuals into catches, observed heterozygosity is also lower than expected, suggests that one of the three possible explanations should be also acting at the catch level. In this sense, the possibilities of inbreeding and non-random sampling members of the same family should also be considered. Indeed, the fact that size distributions are quite homogeneous within catches (**Figure 1**), supports previous observations that individuals are grouped according to their age (Staby et al., 2011), and claims for more studies linking the reproductive behavior of the species and the genetic diversity results obtained here, which should be further confirmed with additional data. Similarly, no explanation to the different genetic diversity estimates obtained per catch could be found. These differences could be simply due to a low sample size per catch or have a more biologically meaningful information, and thus, more analyses are needed to confirm either one.

Overall, we have validated the RAD-seq based SNP discovery method using the *SbfI* restriction enzyme for *M. muelleri*, which has resulted in the first genomic resources for this species that are moreover made available for future studies (raw sequences are available at NCBI SRA Bioproject PRJNA417219). This data will contribute to shed light on phylogeographic patterns within the species (Rodríguez-Ezpeleta et al., 2016), detect genetic adaptation (Van Wyngaarden et al., 2017), and to resolve the phylogeny within the genus for species delimitation (Diaz-Arce et al., 2016).

## Author Contributions

NR-E designed the study, performed the analyses, interpreted the data, and wrote the manuscript. PÁ collected the samples, contributed to the interpretation of the data and revised the manuscript. XI contributed to the interpretation of the data and revised the manuscript.

## Conflict of Interest Statement

The authors declare that the research was conducted in the absence of any commercial or financial relationships that could be construed as a potential conflict of interest.

## References

[B1] Allendorf F. W. Hohenlohe P. A. Luikart G. (2010). Genomics and the future of conservation genetics. *Nat. Rev. Genet.* 11 697–709. 10.1038/nrg2844 20847747

[B2] Amores A. Catchen J. Ferrara A. Fontenot Q. Postlethwait J. H. (2011). Genome evolution and meiotic maps by massively parallel DNA sequencing: spotted gar, an outgroup for the teleost genome duplication. *Genetics* 188 799–808. 10.1534/genetics.111.127324 21828280 PMC3176089

[B3] Baird N. A. Etter P. D. Atwood T. S. Currey M. C. Shiver A. L. Lewis Z. A. (2008). Rapid SNP discovery and genetic mapping using sequenced RAD markers. *PLOS ONE* 3:e3376. 10.1371/journal.pone.0003376 18852878 PMC2557064

[B4] Benestan L. Gosselin T. Perrier C. Sainte-Marie B. Rochette R. Bernatchez L. (2015). RAD genotyping reveals fine-scale genetic structuring and provides powerful population assignment in a widely distributed marine species, the American lobster (*Homarus americanus*). *Mol. Ecol.* 24 3299–3315. 10.1111/mec.13245 25977167

[B5] Bradbury I. R. Laurel B. Snelgrove P. V. Bentzen P. Campana S. E. (2008). Global patterns in marine dispersal estimates: the influence of geography, taxonomic category and life history. *Proc. Biol. Sci.* 275 1803–1809. 10.1098/rspb.2008.0216 18445556 PMC2587791

[B6] Catchen J. Bassham S. Wilson T. Currey M. O’Brien C. Yeates Q. (2013a). The population structure and recent colonization history of Oregon threespine stickleback determined using restriction-site associated DNA-sequencing. *Mol. Ecol.* 22 2864–2883. 10.1111/mec.12330 23718143 PMC3712868

[B7] Catchen J. Hohenlohe P. A. Bassham S. Amores A. Cresko W. A. (2013b). Stacks: an analysis tool set for population genomics. *Mol. Ecol.* 22 3124–3140. 10.1111/mec.12354 23701397 PMC3936987

[B8] Corander J. Majander K. K. Cheng L. Merila J. (2013). High degree of cryptic population differentiation in the Baltic Sea herring *Clupea harengus*. *Mol. Ecol.* 22 2931–2940. 10.1111/mec.12174 23294045

[B9] Cowen R. K. Paris C. B. Srinivasan A. (2006). Scaling of connectivity in marine populations. *Science* 311 522–527. 10.1126/science.1122039 16357224

[B10] Davey J. W. Hohenlohe P. A. Etter P. D. Boone J. Q. Catchen J. M. Blaxter M. L. (2011). Genome-wide genetic marker discovery and genotyping using next-generation sequencing. *Nat. Rev. Genet.* 12 499–510. 10.1038/nrg3012 21681211

[B11] Diaz-Arce N. Arrizabalaga H. Murua H. Irigoien X. Rodriguez-Ezpeleta N. (2016). RAD-seq derived genome-wide nuclear markers resolve the phylogeny of tunas. *Mol. Phylogenet. Evol.* 102 202–207. 10.1016/j.ympev.2016.06.002 27286653

[B12] Earl D. A. vonHoldt B. M. (2012). STRUCTURE HARVESTER: a website and program for visualizing STRUCTURE output and implementing the Evanno method. *Conserv. Genet. Resour.* 4 359–361. 10.1007/s12686-011-9548-7

[B13] Etter P. D. Bassham S. Hohenlohe P. A. Johnson E. A. Cresko W. A. (2011). SNP discovery and genotyping for evolutionary genetics using RAD sequencing. *Methods Mol. Biol.* 772 157–178. 10.1007/978-1-61779-228-1_9 22065437 PMC3658458

[B14] Evanno G. Regnaut S. Goudet J. (2005). Detecting the number of clusters of individuals using the software STRUCTURE: a simulation study. *Mol. Ecol.* 14 2611–2620. 10.1111/j.1365-294X.2005.02553.x 15969739

[B15] Gjøsætr J. Kawaguchi K. (1980). *A Review of the World Resources of Mesopelagic Fish*. Rome: Food and Agriculture Organization 1–151.

[B16] Gordeeva N. V. (2011). On structure of species in pelagic fish: the results of populational-genetic analysis of four species of lanternfish (Myctophidae) from the southern Atlantic. *J. Ichthyol.* 51 152 10.1134/s0032945211020032

[B17] Gordeeva N. V. (2014). Phylogeography, genetic isolation, and migration of deep-sea fishes in the South Atlantic. *J. Ichthyol.* 54 642–659. 10.1134/s003294521406006x

[B18] Gunawickrama K. B. S. Naevdal G. (2001). Genetic and morphological stock structure of the pearlside, *Maurolicus muelleri* (Pisces, Sternoptychidae), among Norwegian fjords and offshore area. *Sarsia* 86 191–201. 10.1080/00364827.2001.10420475

[B19] Haasl R. J. Payseur B. A. (2011). Multi-locus inference of population structure: a comparison between single nucleotide polymorphisms and microsatellites. *Heredity* 106 158–171. 10.1038/hdy.2010.21 20332809 PMC2892635

[B20] Habib K. A. Oh J. Kim S. Lee Y.-H. (2012). Divergence and gene flow between the East Sea and the Southeast Atlantic populations of North Pacific light fish *Maurolicus japonicus* Ishikawa. *Genes Genomics* 34 609–618. 10.1007/s13258-012-0059-z

[B21] Hauser L. Carvalho G. R. (2008). Paradigm shifts in marine fisheries genetics: ugly hypotheses slain by beautiful facts. *Fish Fish.* 9 333–362. 10.1111/j.1467-2979.2008.00299.x

[B22] Hess J. E. Campbell N. R. Close D. A. Docker M. F. Narum S. R. (2013). Population genomics of Pacific lamprey: adaptive variation in a highly dispersive species. *Mol. Ecol.* 22 2898–2916. 10.1111/mec.12150 23205767

[B23] Hulley P. A. Prosch R. M. (1987). Mesopelagic fish derivatives in the southern Benguela upwelling region. *South Afr. J. Mar. Sci.* 5 597–611. 10.2989/025776187784522289

[B24] Irigoien X. Klevjer T. A. Røstad A. Martinez U. Boyra G. Acuña J. L. (2014). Large mesopelagic fishes biomass and trophic efficiency in the open ocean. *Nat. Commun.* 5:3271. 10.1038/ncomms4271 24509953 PMC3926006

[B25] Jakobsson M. Rosenberg N. A. (2007). CLUMPP: a cluster matching and permutation program for dealing with label switching and multimodality in analysis of population structure. *Bioinformatics* 23 1801–1806. 10.1093/bioinformatics/btm233 17485429

[B26] Jombart T. Ahmed I. (2011). adegenet 1.3-1: new tools for the analysis of genome-wide SNP data. *Bioinformatics* 27 3070–3071. 10.1093/bioinformatics/btr521 21926124 PMC3198581

[B27] Kaartvedt S. Staby A. Aksnes D. L. (2012). Efficient trawl avoidance by mesopelagic fishes causes large underestimation of their biomass. *Mar. Ecol. Prog. Ser.* 456 1–6. 10.3354/Meps09785

[B28] Kim S. Kim C. Oh J. Kim B. Seo H. Kim W. (2008). Genetic similarity between the South Atlantic and the western North Pacific *Maurolicus* (Stomiiformes: Actinopterygii) taxa, *M-walvisensis* Parin & Kobyliansky and *M-japonicus* Ishikawa: evidence for synonymy? *J. Fish Biol.* 72 1202–1214. 10.1111/j.1095-8649.2007.01786.x

[B29] Klevjer T. Irigoien X. Røstad A. Fraile-Nuez E. Benítez-Barrios V. Kaartvedt S. (2016). Large scale patterns in vertical distribution and behaviour of mesopelagic scattering layers. *Sci. Rep.* 6:19873. 10.1038/srep19873 26813333 PMC4728495

[B30] Kojima S. Moku M. Kawaguchi K. (2009). Genetic diversity and population structure of three dominant myctophid fishes (*Diaphus theta, Stenobrachius leucopsarus*, and *S. nannochir*) in the North Pacific Ocean. *J. Oceanogr.* 65 187–193. 10.1007/s10872-009-0018-8

[B31] Kristoffersen J. B. Salvanes A. G. V. (2009). Distribution, growth, and population genetics of the glacier lanternfish (*Benthosema glaciale*) in Norwegian waters: contrasting patterns in fjords and the ocean. *Mar. Biol. Res.* 5 596–604. 10.1080/17451000903042479

[B32] Lischer H. E. Excoffier L. (2012). PGDSpider: an automated data conversion tool for connecting population genetics and genomics programs. *Bioinformatics* 28 298–299. 10.1093/bioinformatics/btr642 22110245

[B33] Narum S. R. Buerkle C. A. Davey J. W. Miller M. R. Hohenlohe P. A. (2013). Genotyping-by-sequencing in ecological and conservation genomics. *Mol. Ecol.* 22 2841–2847. 10.1111/mec.12350 23711105 PMC3935057

[B34] Okiyama M. (1971). Early life history of the gonostomatid fish, *Maurolicus muelleri* (Gmelin), in the Japan Sea. *Bull. Japan Sea Reg. Fish. Res. Lab.* 23 21–53.

[B35] Palumbi S. (2003). Population genetics, demographic connectivity, and the design of marine reserves. *Ecol. Appl.* 13 146–158. 10.1890/1051-0761(2003)013[0146:PGDCAT]2.0.CO;2

[B36] Parin N. V. Kobyliansky S. G. (1993). Review for the genus *Maurolicus* (Sternoptychidae, Stomiiformes) with re-establishing validity of five species considered junior synonyms of *M. Mueller* and descriptions of nine species. *Biol. Ocean Fish. Squids* 128 69–101.

[B37] Pritchard J. K. Stephens M. Donnelly P. (2000). Inference of population structure using multilocus genotype data. *Genetics* 155 945–959. 10835412 10.1093/genetics/155.2.945 PMC1461096

[B38] Puebla O. Bermingham E. McMillan W. O. (2014). Genomic atolls of differentiation in coral reef fishes (*Hypoplectrus* spp. Serranidae). *Mol. Ecol.* 23 5291–5303. 10.1111/mec.12926 25231270

[B39] Purcell S. Neale B. Todd-Brown K. Thomas L. Ferreira M. A. Bender D. (2007). PLINK: a tool set for whole-genome association and population-based linkage analyses. *Am. J. Hum. Genet.* 81 559–575. 10.1086/519795 17701901 PMC1950838

[B40] Rašić G. Filipović I. Weeks A. R. Hoffmann A. A. (2014). Genome-wide SNPs lead to strong signals of geographic structure and relatedness patterns in the major arbovirus vector. *Aedes aegypti*. *BMC Genomics* 15:275. 10.1186/1471-2164-15-275 24726019 PMC4023594

[B41] Rees D. J. Byrkjedal I. Sutton T. T. (2017). Pruning the pearlsides: reconciling morphology and molecules in mesopelagic fishes (*Maurolicus*: Sternoptychidae). *Deep Sea Res. II Top. Stud. Oceanogr.* 137(Suppl. C) 246–257. 10.1016/j.dsr2.2016.04.024

[B42] Robertson A. (1965). The interpretation of genotypic ratios in domestic animal populations. *Anim. Sci.* 7 319–324. 10.1017/S0003356100025770 12925896

[B43] Robertson D. A. (1976). Planktonic stages of *Maurolicus muelleri* (Teleostei Sternoptychidae) in New Zealand waters. *N. Z. J. Mar. Freshw. Res.* 10 311–328. 10.1080/00288330.1976.9515615

[B44] Rodríguez-Ezpeleta N. Bradbury I. R. Mendibil I. Álvarez P. Cotano U. Irigoien X. (2016). Population structure of Atlantic mackerel inferred from RAD-seq-derived SNP markers: effects of sequence clustering parameters and hierarchical SNP selection. *Mol. Ecol. Resour.* 16 991–1001. 10.1111/1755-0998.12518 26936210

[B45] Rosenberg N. A. (2004). DISTRUCT: a program for the graphical display of population structure. *Mol. Ecol. Notes* 4 137–138. 10.1046/j.1471-8286.2003.00566.x

[B46] Rousset F. (2008). Genepop’007: a complete reimplementation of the Genepop software for Windows and Linux. *Mol. Ecol. Resour.* 8 103–106. 10.1111/j.1471-8286.2007.01931.x 21585727

[B47] St. John M. A. Borja A. Chust G. Heath M. Grigorov I. Mariani P. (2016). A dark hole in our understanding of marine ecosystems and their services: perspectives from the mesopelagic community. *Front. Mar. Sci.* 3:31 10.3389/fmars.2016.00031

[B48] Staby A. Røstad A. Kaartvedt S. (2011). Long-term acoustical observations of the mesopelagic fish *Maurolicus muelleri* reveal novel and varied vertical migration patterns. *Mar. Ecol. Prog. Ser.* 441 241–255. 10.3354/meps09363

[B49] Sutton T. (2013). Vertical ecology of the pelagic ocean: classical patterns and new perspectives. *J. Fish Biol.* 83 1508–1527. 10.1111/jfb.12263 24298949

[B50] Szulkin M. Gagnaire P. A. Bierne N. Charmantier A. (2016). Population genomic footprints of fine-scale differentiation between habitats in Mediterranean blue tits. *Mol. Ecol.* 25 542–558. 10.1111/mec.13486 26800038

[B51] Van de Putte A. P. Van Houdt J. K. J. Maes G. E. Hellemans B. Collins M. A. Volckaert F. A. M. (2012). High genetic diversity and connectivity in a common mesopelagic fish of the Southern Ocean: the myctophid *Electrona antarctica*. *Deep Sea Res. II Top. Stud. Oceanogr.* 59(Suppl. C) 199–207. 10.1016/j.dsr2.2011.05.011

[B52] Van Wyngaarden M. Snelgrove P. V. DiBacco C. Hamilton L. C. Rodriguez-Ezpeleta N. Jeffery N. W. (2017). Identifying patterns of dispersal, connectivity and selection in the sea scallop, *Placopecten magellanicus*, using RADseq-derived SNPs. *Evol. Appl.* 10 102–117. 10.1111/eva.12432 28035239 PMC5192885

[B53] Wahlund S. (1928). Zusammensetzung von populationen und korrelationserscheinungen vom standpunkt der vererbungslehre ausbetrachtet. *Hereditas* 11 65–106. 10.1111/j.1601-5223.1928.tb02483.x

[B54] Ward R. D. Woodmark M. Skibinski D. (1994). A comparison of genetic diversity levels in marine, freshwater and anadromous fishes. *J. Fish Biol.* 44 213–232. 10.1111/j.1095-8649.1994.tb01200.x

[B55] Weir B. S. Cockerham C. C. (1984). Estimating F-statistics for the analysis of population structure. *Evolution* 38 1358–1370. 28563791 10.1111/j.1558-5646.1984.tb05657.x

[B56] Wright S. (1921). Systems of mating II. The effects of inbreeding on the genetic composition of a population. *Genetics* 6 124–143. 17245959 10.1093/genetics/6.2.124 PMC1200502

